# Multifunctional leather finishing vs. applications, through the addition of well-dispersed flower-like nanoparticles

**DOI:** 10.1038/s41598-024-51775-4

**Published:** 2024-01-25

**Authors:** Francesca Fierro, Mariagrazia Iuliano, Claudia Cirillo, Claudia Florio, Gaetano Maffei, Andrea Loi, Todor Batakliev, Renata Adami, Maria Sarno

**Affiliations:** 1 Department of Physics “E.R. Caianiello”, University of Salerno, Via Giovanni Paolo II, 132, 84084 Fisciano, SA Italy; 2 Centre NANO_MATES, University of Salerno, Via Giovanni Paolo II, 132, 84084 Fisciano, SA Italy; 3 Department of Industrial Engineering, University of Salerno, Via Giovanni Paolo II, 132, 84084 Fisciano, SA Italy; 4 Stazione Sperimentale per l’Industria delle Pelli e delle materie concianti-SSIP (Italian National Leather Research Institute), Comprensorio Olivetti, Via Campi Flegrei, 34, 80078 Pozzuoli, NA Italy; 5 Conceria DMD SOLOFRA Spa, Via Celentane, 9, 83029 Solofra, AV Italy; 6 Mario Levi Italia s.r.l., Via Arzignano, 130, 36072 ChiampoVI, Italy; 7 Open Laboratory on Experimental Micro and Nano Mechanics (OLEM), Institute of Mechanics, Bulgarian Academy of Sciences, Acad. G. Bonchev Str., Block 4, 1113 Sofia, Bulgaria

**Keywords:** Nanoscale materials, Chemistry, Engineering, Materials science, Nanoscience and technology

## Abstract

In the present paper, multifunctional flower-like nanoparticles were synthesized to be used in the leather finishing. They are capable of conferring simultaneously and synergistic antimicrobial, self-cleaning, light resistance, hydrophobic, mechanical, thermal, and fluorescent properties due to the presence of Ag, TiO_2_, and SiO_2_ NPs. These nanoparticles form a “flower-like” structure in which the “pistil” is made up of TiO_2_ and the “petals” that surround it of silver nanoparticles and silica nanoparticles, whose dimensions are of the order of ten nanometers. Their surfaces enjoy abundant hydrophilic functionalities to be dispersed within inks commonly used during the leather finishing process. Leathers functionalized with these nanomaterials showed significantly improved self-cleaning properties after 15 h of exposure to UV light, and antibacterial properties 10 times higher than that shown by the untreated samples. Aging tests were performed (ISO 105-B02, ISO 17228, SAEJ 2412). ΔE, color variation decreased by approximately 30%, if compared with samples not refined with flower-like NPs. Furthermore, the results of the mechanical tests (ISO 17076, FCA 50444) evidence amazing properties, e.g. abrasion resistance more than significantly improved, increase in resistance from 1500 cycles for the untreated samples to 3000 cycles for the leathers finished with flower-like NPs. The contact angle analysis, capturing the angle that traces the air–water to water–substrate interface from the origin of the air–water-substrate contact point at the edge, is practically unchanged after 10 s in the case of nanoparticles containing finishing.

## Introduction

Leather is one of the most used and marketed raw materials worldwide and one of the oldest examples of circular economy. This is attributed to its incredibly versatile nature and variety of applications, for which it has progressively received more and more attention. The leather and leather products industry plays a leading role in the world economy. The Italian tanning industry is historically considered a world leader in terms of value (63% at the EU level, 24% of the world total).

Before taking on its definitive appearance, the leather undergoes numerous processing phases. During the tanning, conservation, and dyeing phases, some characteristics such as softness, mechanical resistance, and color are outlined, while, during the important finishing phase, the aesthetic and functional performance of the leather is defined, protecting, and ennobling its surface. Generally, finishing operations can be divided into mechanical processing and the application of surface coatings in which a finishing film is created. The finishing matrix is generally made up of polymers, which have the task of providing the desired functional characteristics, based on the type of finished leather. Although the leather industry holds a significant place in the global economy, the scarcity of raw hides and skins, competitive leather substitutes, growing ethical concerns, and new manufacturing regulations are now bottlenecks for growth^1^. These issues can be partly solved by nanotechnology, e.g. new efficient tannery effluent treatment and innovative products. Nanomaterials have been applied at various stages of leather manufacturing to achieve better performances. Nowadays, inorganic nanomaterials such as metals/metals oxides have received great attention in leather finishing^1,2^, to confer different properties, such as increased thermal stability, protection against micro-organisms, as well as self-cleaning properties, etc.…, leading to increased leather versatility.

In this regard, in numerous studies, it has been underlined how the surface finishing of leather with TiO_2_ and SiO_2_ NPs shows self-cleaning and thermal resistance behavior^3,4^. Low-cost, environmentally friendly, and stable titanium dioxide is considered an extremely promising photocatalyst. Indeed, under UV/visible irradiation, generated radical species take part in oxidation reactions destroying organic contaminants, inactivating microorganisms, and increasing heat resistance^5^. However, due to the large band gap, 3.20 eV, only a small UV fraction of sunlight, about 4–6%, is usable. To overcome this problem, studies have been performed adopting a variety of different approaches, mainly based on TiO_2_ doping^6,7^, and also to shift adsorption in the visible region. For example, it is well known that doping TiO_2_ with silica can significantly increase its photocatalytic activity^8–15^. Silica can influence thermal stability, too^16^. Silica-based materials have been found promising in improving thermal stability, which is strongly dependent on the dispersion of SiO_2_ nanoparticles. This is attributable to the trapping effect of polymeric radicals by silica particles together with hydrogen bonds between the polymer matrix and the OH groups of silica.

Inorganic nanomaterials such as metals/metals oxides have been demonstrated suitable to confer other relevant properties such as protection against UV radiation and brilliance of the surfaces^17^. On the other hand, in this context, the photofading and photostability of dyed and pigmented polymers, occurring when a dye or pigment sensitizes or accelerates the breakdown in the polymer molecular structure, inducing molecular weight reduction or possibly even crosslinking of the polymer and yellowing, is a topic of considerable interest but little explored. Indeed, these phenomena, which are typically accelerated by environmental conditions, temperature, humidity, oxygen, and UV content of the light source, are also induced by the presence of photoactive dyes and pigments and chemical bonding involving colorants and polymer matrix.

Another aspect that can be influenced is the bacteria growth on the leather surface favored by atmospheric humidity, water permeability, temperature, and the presence of oxygen which can cause the formation of bad smell, microbial proliferation, and poor resistance to abrasion and discoloration. Different metals and metal oxides have been suggested and studied for their bactericidal capability^18,19^. Metallic nanoparticles, such as Ag, are popular nano anti-microbial agents already used in various consumer products^20^, also in combination with other nanoparticles, e.g., with TiO_2_^21,22^. In addition, Ag NPs are less toxic to humans as compared to other metals and they have been widely selected as suitable antibacterial finishing agents for textile fibers^23–31^. In recent years, silver-based semiconductor materials have been considered candidates for photocatalysis applications owing to their suitable band gap and good structural stability^32,33^, too.

The incorporation of different nanoparticles into the finishing layer can also lead to an improvement in the mechanical properties and strong adhesion to the leather^4,17^.

In the literature, typically, one or more functionalities, have been explored^1,34^, showing anyway interesting performance for functional leather finishes, sometimes more significant, other times less so. More generally, real industrial interest is still far away, industrial research is limited, and the studies are far from systematic because of tangible applications. Several issues remain firstly, stability and non-irreversible loss of dispersion stability during storage or transportation; systematic studies that privilege leather finishes with diverse functions; research to reveal the relationships between the microstructures and species combinations on the performance of finished leathers.

In this study, considering the possibility of inducing superior efficacy of the finishing coating towards a series of multifunctional properties, by adding nanoparticles, flower-shaped NPs, where “pistil” and “petals” give distinct functions, were prepared. This approach allows not only to enjoy different NP properties but also enhancement/amplification of these properties due to the heterojunctions between nanoparticles. To encourage homogeneous finishing film deposition, here in a one-step addition for simultaneous antimicrobial, mechanical, and thermal properties, as well as UV and water protection, and photocatalytic activity, particular attention has been paid to the dispersion in the finishing material. The nanoparticles were prepared through a simple and scalable synthetic approach, which yields mass production of nanoparticles with organic molecules functionalized surfaces to be appropriately dispersed in finishing polymers and applied on sheep, goat, and bovine leathers, for footwear/leather goods and the automotive sector.

In particular, a three-dimensional flower-shaped structure, enjoying heterojunctions, e.g. between TiO_2_ and SiO_2_ favouring photocatalysis, between Ag and TiO_2_ to inhibit microbial growth, … and overall containing SiO_2_ nanoparticles to improve poisoning molecules adsorption, specific surface area^35^, abundant surface hydroxyl groups, was studied for antimicrobial, mechanical, and thermal properties, as well as UV protection and the photocatalytic activity.

In this study, the aspects of safety and sustainability, including economic sustainability, from the laboratory scale to industrial research, were taken care of. Moreover, it started in 2019 in a context of close cooperation between industry and scientific research, allowing even more crucial importance because of the COVID-19 event. Indeed, though it has proved disastrous for the economy, it has inculcated an awareness among the people to use bio-safe materials. In this sense, for the leather industry, a consumer-focused sector^36^, these results are much more significant and perspective.

## Experimental

### Materials

For the synthesis of the flower-like (FL) nanoparticles (NPs), the following were used: deionized water and methanol, silver nitrate (AgNO_3_, Sigma Aldrich > 99), titanium isopropoxide (Sigma Aldrich ≥ 99.9), tetraethyl orthosilicate (TEOS, Sigma Aldrich > 99 %), (3-Aminopropyl)triethoxysilane (APTES), cyclohexane, ammonia hydroxide, oleic acid, 1,2 hexadecanediol, benzyl ether, ethanol, and other chemicals were acquired from Sigma Aldrich. All chemicals were of analytical grade.

### Synthesis of flower-like nanoparticles

The synthesis of flower-like nanoparticles took place in several steps: firstly, titania nanoparticles were made by chemical precipitation method; these were subsequently added to the synthesis of silver nanoparticles, which was performed by thermal decomposition method and finally, the obtained nanoparticles were introduced into the synthesis of silica nanoparticles, which was performed by a microemulsion method. Fluorescent molecules such as organic dyes could be directly conjugated to silica nanoparticles. The syntheses were carried out in reaction volumes, heated by thermoresistances, with a capacity of around 5 L, for a production of 2–4 g/day, sufficient to meet the needs of the leather production lines.

#### Synthesis of TiO_2_ nanoparticles

To perform the synthesis of TiO_2_ NPs, the following steps were performed: titanium isopropoxide was slowly added dropwise in deionized water (0.62 mL titanium isopropoxide/water). Subsequently, the solution was mixed by continuous stirring at 40 °C for 30 min. At the end of this step, a white precipitate of TiO_2_ NPs was obtained at the bottom of the beaker. The precipitate was separated from the mixture by centrifugation and washed several times with cycles of deionized water and methanol. Then, the precipitate was dried at 80 °C for 12 h and calcined at 400 °C in the air for 2 h.

#### Synthesis of Ag-TiO_2_ nanoparticles

For the synthesis of Ag-TiO_2_ nanoparticles, the following reagents were used: 10 mg/mL of TiO_2_ NPs, 0.1 mmol/mL of silver nitrate, 0.5 mmol/mL of 1,2-hexadecanediol, 0.6 mmol/mL of oleic acid in benzyl ether. All reagents were mixed in a batch reactor and stirred magnetically under nitrogen flow from 2 to 200 °C for 2 h and, subsequently, at 285 °C for 1 h. After the synthesis, the produced mixture was washed through centrifugation cycles (7500 rpm; 30 min) in ethanol and hexane 3–4 times. After, the produced material was left to dry for 24 h at room temperature.

#### Synthesis of flower-like nanoparticles (Ag-TiO_2_-SiO_2_)

To carry out the preparation of Ag-TiO_2_-SiO_2_ nanoparticles with fluorescent agents, a conjugate consisting of fluorescein isothiocyanate (FITC) and (3-Aminopropyl)triethoxysilane (APTES) was prepared in a 2:1 molar ratio 5 μL/mL in ethanol. The mixture was kept in the dark under stirring for 4 h; finally, the reaction product, consisting of the FITC-APTES conjugate, was stored at 4 °C. At this point, the real synthesis of the flower-like NPs takes place. In particular, a microemulsion of Igepal CO-520 and cyclohexane has been prepared. Subsequently, the FITC-APTES conjugate and approximately 1 μL/mL Ag-TiO_2_ NPs/hexane were added to the previously prepared microemulsion. Then 0.83 μL/mL tetraethoxysilane (TEOS)/ammonia hydroxide was added. After 24 h, necessary for hydrolysis and condensation of the silica precursor, the flower-shaped nanoparticles with fluorescent agents were separated from the mixture by centrifugation.

### Nanoparticles characterization

#### Chemical-physical characterization

Nanoparticles were structurally and morphologically characterized using the following techniques: X-ray diffraction (XRD) measurements were performed by a Bruker D2 X-ray diffractometer using CuKα radiation.

Scanning Electron Microscope (SEM) images were obtained with a TESCAN-VEGA LMH; 230 V coupled with an Energy Dispersive X-ray Spectroscopy (EDS) probe. The samples, without any pre-treatment, were covered with a 50 Å thick chromium film using a sputter coater (QUORUM 150 T).

Transmission Electron Microscope (TEM) images were obtained with an FEI Tecnai microscope working at 200 keV, with a LaB_6_ filament as the source of electrons, equipped with an energy-dispersive X-ray spectroscopy (EDX) probe. For the preparation of the TEM samples, drops of nanoparticles suspension in ethanol were deposited on carbon-coated electron microscope grids.

Nanoparticles Tracking Analysis (NTA) measurements were performed on the nanoparticles with a Malvern NanoSight LM10-Malvern Instruments. Such analysis allows for determining the profile of the size distribution of small particles suspended in a liquid. The technique is used in combination with an ultramicroscope which allows for visualization of the movement of particles in liquid suspension under the effect of Brownian motion. The computer software tracks the motions of the particles and estimates their hydrodynamic radius using the Stokes–Einstein equation.

For the thermogravimetric studies (TG-DTG), TGA 2 METTLER TOLEDO was used under an air flow at 10 °C/min. FT-IR spectra were obtained by Nicolet iS50 FT-IR. Fluorescence was recorded on a Varian Cary Eclipse spectrophotometer.

#### Characterization of nanoparticle properties in finishing inks

##### Nanoparticles fluorescence

To verify the fluorescence of the nanoparticles, a UV light, with a wavelength λ = 365 nm compliant with those used to verify the anti-counterfeiting of materials, in a dark chamber, was used. UV–Vis measurements were performed in a 1 cm quartz cuvette using water as the solvent on a Cary 50, Varian, UV–Vis spectrophotometer. Fluorescence spectra were recorded using a 1 cm quartz cuvette, water as a solvent on a Cary Eclipse spectrophotometer (Varian), by excitation of 500 to 800 nm.

##### Dispersion of nanoparticles in finishing inks

For leathers finishing with flower-like nanoparticles, they were dispersed in polyurethane-based (code 5766, RODA FIX 5766 GLOSS, TFL Italia S.p.A.) and nitroemulsion (code 707, FYL WAX 707, GSC GROUP S.p.A.) finishing inks using an ultrasound tip (Hielscher UP400S) at the maximum power of ultrasounds for 10 min. Subsequently, the finishing inks were applied to the leather by spraying in industrial paint booths by specialized workers, scanning horizontally and then vertically 4 times.

### Leather finishing characterization

The leather samples finished with nanoparticles are listed in Table 1. The finishing chemicals were mixed with the prepared nanoparticles at different concentrations (mg/mL), also thanks to the use of an ultrasound tip.

**Table 1:** Samples of leather with flower-like nanoparticles.

Samples	Composition of the mixture Flower-Like (FL). FL_x_ (x = mg/L	Finished leather according to color	Finishing chemical
A	FL_10_	Pink	707
B	FL_10_	Pink	5766
C	FL_10_	White	707
D	FL_10_	White	5766
P (control)	None	Pink	None
W (control)	None	White	None

#### Chemical-physical characterization

The leather samples without finishing and with finishing (flower-like NPs in 5766 and 707 inks) were characterized from the chemical-physical point of view by Scanning Electron Microscope (SEM) whose images were obtained with a TESCAN-VEGA LMH; 230 V coupled with an energy dispersive X-ray spectroscopy (EDS) probe. Such analysis allows us to determine the profile of the size distribution of small particles on a surface. For thermogravimetric studies (TG-DTG), on the other hand, METTLER TOLEDO TGA 2 was used under an air flow at 10 °C/min, finally, the FT-IR spectra were obtained from Nicolet iS50 FT-IR.

#### Leather finishing properties characterization details

- **Self-cleaning properties under UV–vis light:** the nanoparticle-finished leathers were stained with a drop of methylene blue dye (C_16_H_18_ClN_3_S_3_H_2_O, MB) at a concentration of 20 ppm and exposed to UV light (irradiance at λ = 365 nm). The change in the color of the spot was evaluated over time by photography.
- **Aging tests**: for the samples shown in Table 1, accelerated tests of resistance to sunlight were carried out after a typical finishing procedure (DMD SOLOFRA spa—Solofra (AV), Italy). In particular, a paint spray gun (5 atm) loaded with 10 mL of the finishing mixtures was used on each leather to ensure coverage of the entire surface. Once dried, the samples were cut out, placed on a black card, and subjected to a xenon lamp (Solarbox 1500 standard) at a temperature of 50 °C for 24 h and 48 h. For comparison, a portion of the sprayed samples was covered to avoid exposure to the xenon lamp. The method for evaluating the discoloration of sheep and goat leathers for shoes and bags is associated with the ISO 105 B02 standard. The accelerated aging tests were conducted on samples of sheep and bovine leather for the various intended uses (respectively footwear and leather goods). These tests made it possible to verify the color fastness of the leather samples after having subjected them to 60 °C in an oven for 72 h, according to the ISO 17228 standard, method 6C. UNI EN ISO 17228 method 6c specifies various aging procedures to obtain an indication of the changes that could occur when the skin is exposed to a certain environment for a prolonged time. The procedures proposed by the standard involve exposing a skin sample to at least one of the following three conditions: heat; heat and humidity; different temperature and humidity cycles.

The test conditions to be used depend on the type of leather and its intended use.

The aging test was also conducted on leather samples for the automotive industry (Mario Levi Italia srl—Chiampo (VI), Italy). These tests were performed according to the SAE J-2412 method which involves exposing the samples to UV rays and visible light generated by a xenon arc inside a controlled radiation chamber. The method also includes a dark cycle under high humidity conditions. This method was performed at an irradiance level of 225 KJ/m^2^, 601 KJ/m^2,^ and 1240 KJ/m^2^.

(iii) ** Antibacterial tests**: antimicrobial properties were also tested by TTC/MALT dipslide tests; in this case, samples of 20 cm^2^ large, from leathers for automotive and leather goods, were impregnated (grain side) with the matrix of the culture media mentioned above. The dipslides with the contaminated culture media were then heated in an oven at 36 °C, to evaluate the growth of the bacterial population, CFU/mL (Colony Forming Units/mL), at 24, 48, and 72 h.
(iv) **Abrasion resistance tests and wear and micro scratch measurements:** abrasion resistance tests were performed on the automotive leather samples by ISO 17076 part 1 (Taber Method) and part 2 (Martindale ball plate). Considering that the application of the finishing preparation was carried out on a laboratory scale, without the use of a pre-primer, the samples were subjected to one hundred cycles, a lower number than those required by the regulations/specifications.

The micro-scratching and wear experiments were performed on the UMT-2 tribometric system (Bruker, USA). Each micro-scratch test was performed using a diamond conical stylus with a tip radius of 5 μm and sliding it under an increasing linear load from 50 to 300 mN. The wear test, on the other hand, was performed with the alternating movement of a chromium stainless steel ball (diameter 6.35 mm) on the surface of the specimen. The normal force applied is 2 N for a time of 300 s.

(e) **Evaluation of the contact angle and absorption dynamics:** the determination of the contact angle θ, i.e. the angle that forms a drop of liquid resting on the solid surface to be examined, is a particularly useful parameter for studying the wettability characteristics of surfaces in more detail. Through this parameter it is, in fact, possible to determine the degree of hydrophobicity and hydrophilicity of the substrate under study: an angle θ < 90° indicates that the surface is hydrophilic; an angle θ > 90° indicates that the surface is hydrophobic.

In this work, this parameter was evaluated through the use of a Pocket Goniometer (Test method D724) which measures the interaction between a drop of water or other liquid and a solid surface over time. With this instrument, through an integrated peristaltic pump, a drop of water is applied to a solid surface; through acquisitions in dynamic mode, it was possible to capture the sequence of images to monitor the interaction of the drop that expands and/or penetrates the material.

(f) **Fluorescence leather:** for fluorescence detection, tests were performed on all functionalized leather. In particular, the finishing inks were applied to the hides by dabbing. After drying at room temperature, the hides were illuminated by a UV lamp with a wavelength λ equal to 365 nm.

## Results and discussion

### Nanoparticles characterization

#### Chemical-physical characterization of nanoparticles

**Figure 1:**
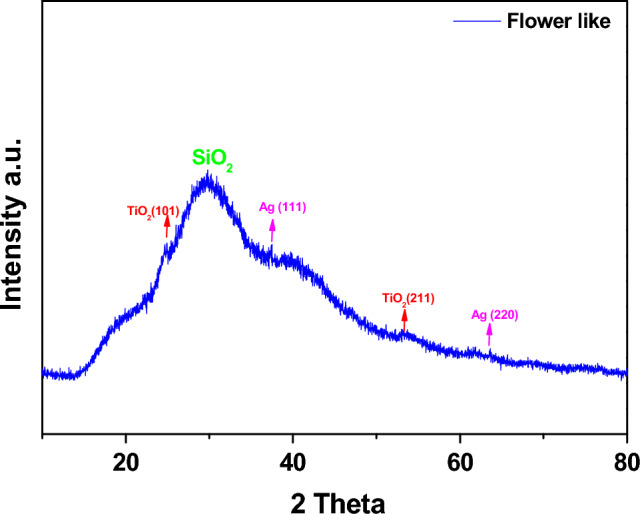
XRD spectrum of flower-like nanoparticles.

The XRD profile of flower-like NPs (Fig. 1) evidences the typical halo broad assigned to amorphous silica. Also, peaks due to titania and silver nanoparticles are present. The Ag peaks can be observed at 38.40° (1 1 1) and 64.78°(2 2 0) (JCPDS card no: 65-2871)^37^. The diffraction peaks of TiO_2_ are well indexed by the anatase phase at 25° (101) and the rutile phase at 55° (211) according to the standard JCPDS database 71-1166 and 73-1763^38^.

**Figure 2:**
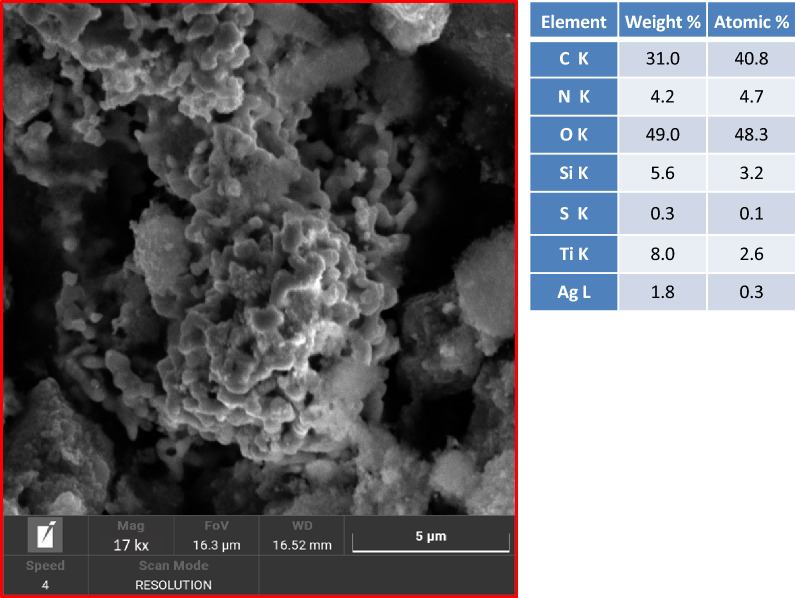
SEM image and EDX data of flower-like nanoparticles.

The morphology of the synthesis product was also analyzed by scanning electron microscopy (SEM). Figure 2 shows an image of the product of the synthesis. The image reveals the presence of structures with sizes up to 1 µm. The characteristics of the powder and the instrument resolution do not allow to distinguish the single nanoparticles, see the following TEM and NTA characterizations.

**Figure 3:**
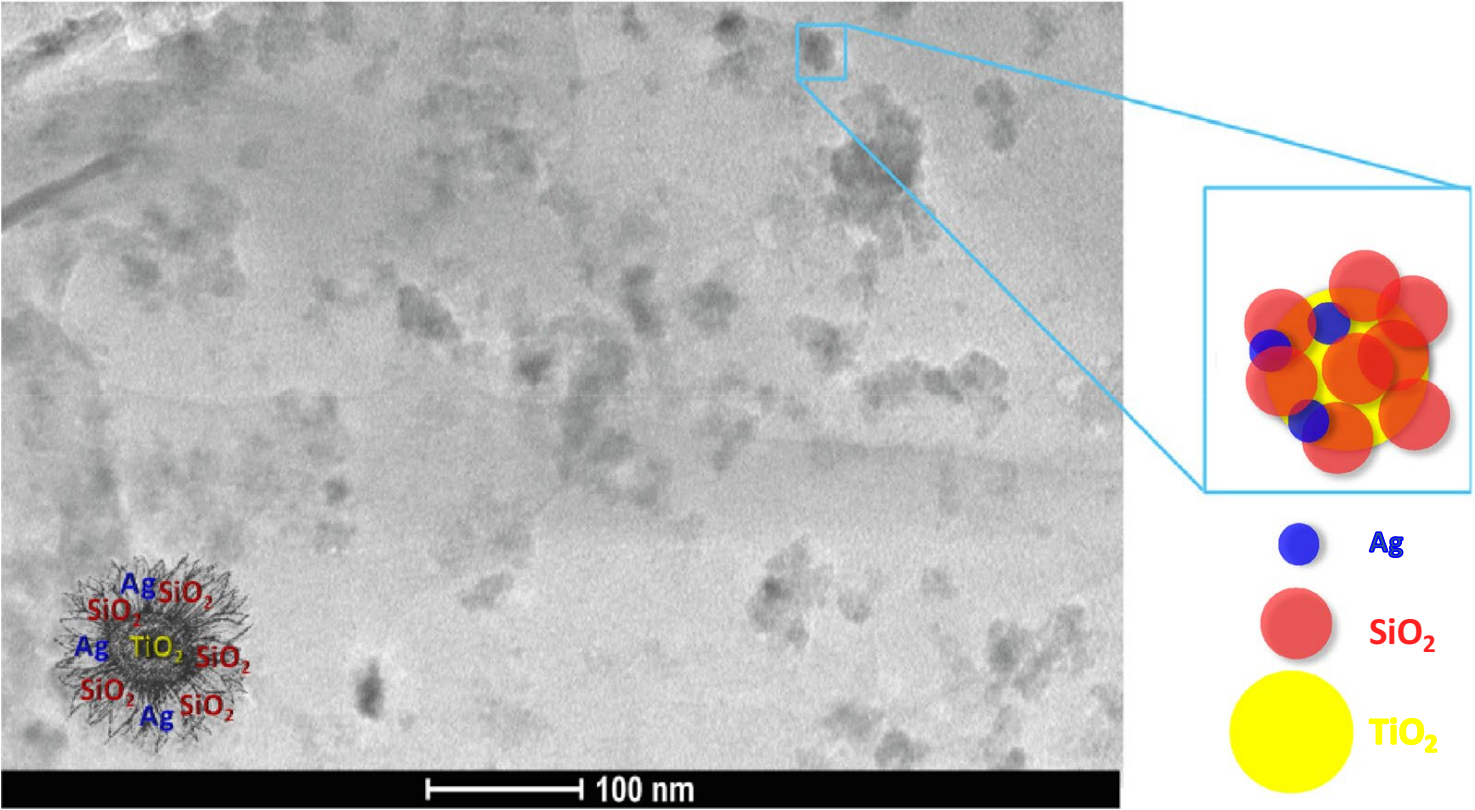
TEM image of flower-like nanoparticles.

Figure 3 shows a TEM image of the product after the synthesis (flower-like sample). The image revealed the formation of nanoparticles of uniform size (approximately 65–70 nm in diameter), which are constituted by a TiO_2_ core (“pistil”) and, various lower size silica and silver NPs “petals” in new nano-sized particles that exhibit structural similarity to plant flowers.

**Figure 4:**
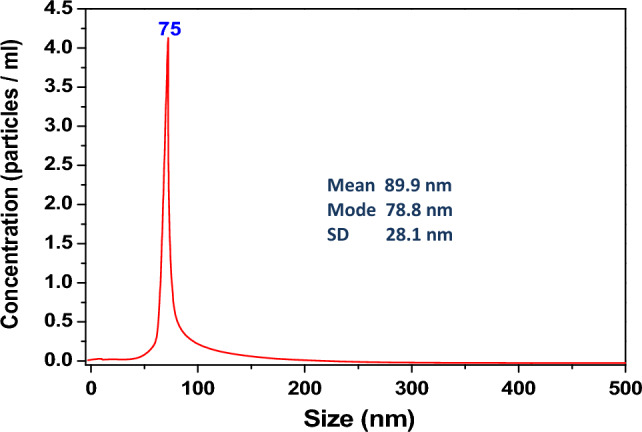
NTA analysis of flower-like nanoparticles.

In Fig. 4, the result of the NTA analysis performed on the nanoparticles is shown. Such an analysis allows us to determine the profile of the size distribution of small particles suspended. The analysis of the size distribution obtained by NTA shows that the nanoparticles sizes are centered at about 75 nm.

**Figure 5:**
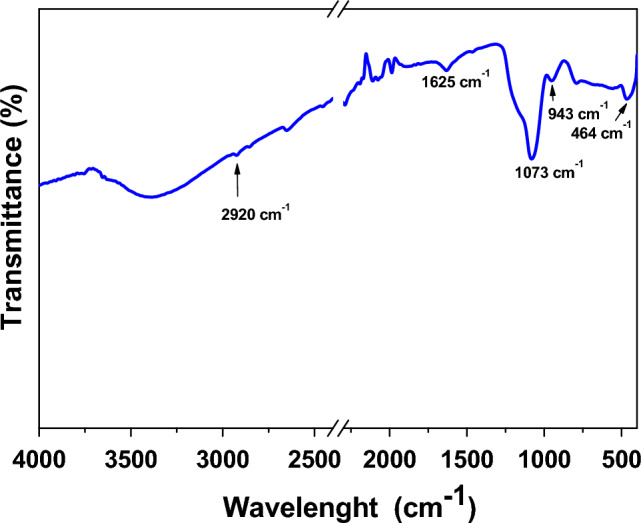
FTIR spectra of flower-like nanoparticles.

In Fig. 5 it is possible to observe the FT-IR spectrum of the flower-like nanoparticles. The profile shows a vibrational band around 1082/cm due to the asymmetric stretching of the Si–O–Si group, the two vibrational bands at 947/cm are due to the asymmetric bending stretching of the Si–OH bond. The vibrational band at 469/cm is due to the vibrations of the Ti–O–Ti bond, while the vibrational band around 1635/cm is due to the Ti–OH bond. The vibrational bands around 2923/cm can be attributed to the presence of secondary amines in the FITC molecule.

#### Characterization of nanoparticle properties

- Nanoparticles fluorescence

**Figure 6:**
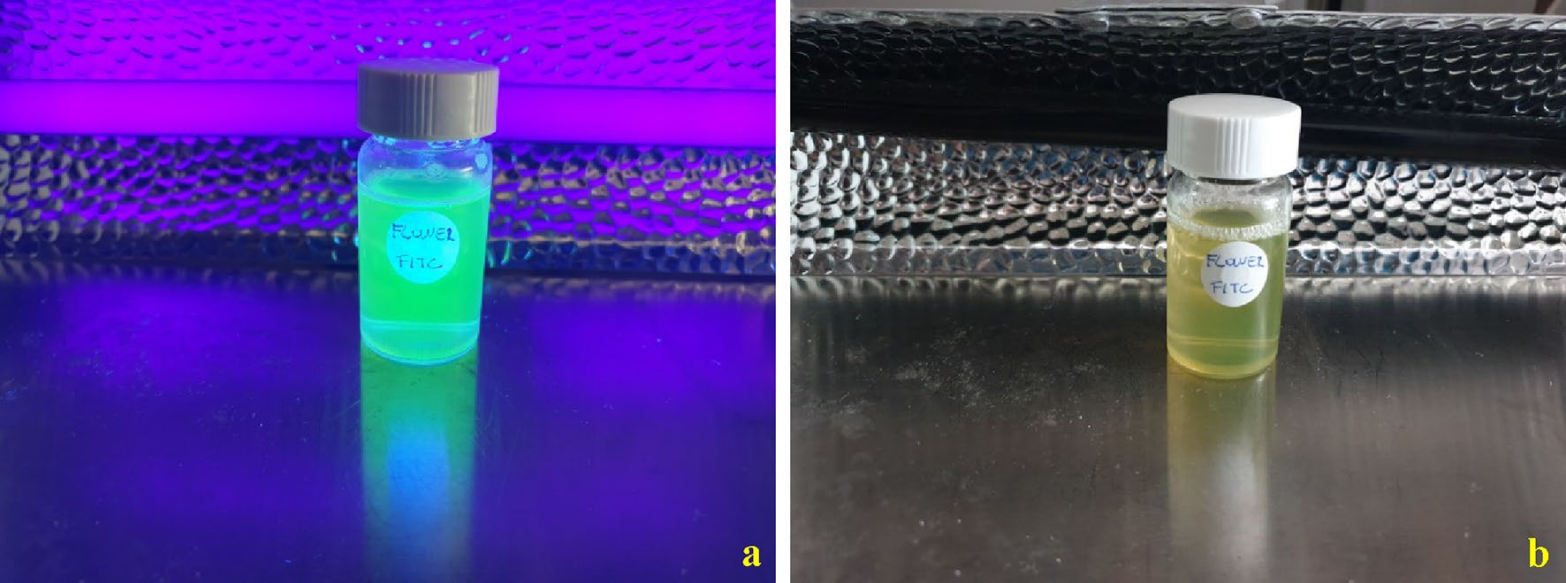
Photographs of flower-like nanoparticles, containing FITC, dispersed in water: under UV lamp (**a**); and, before UV exposition (**b**).

**Figure 7:**
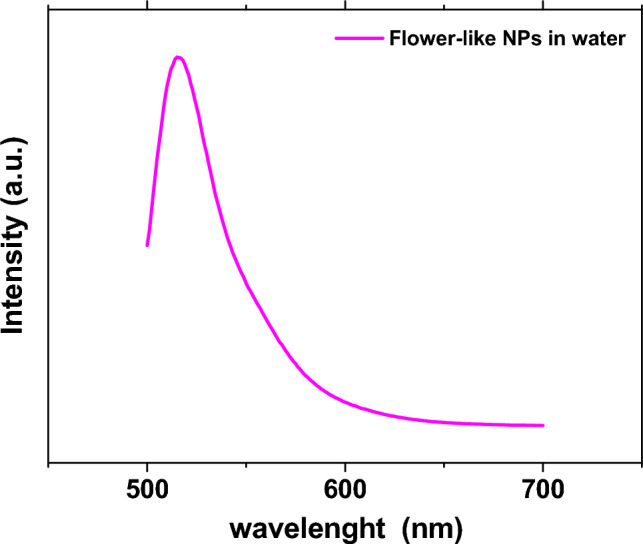
Fluorescence spectrum of flower-like NPs.

In Fig. 6, photographs of flower-like nanoparticles, containing FITC, dispersed in water: before UV exposition and, under a UV lamp are shown. It is possible to observe how nanoparticles, once synthesized, are perfectly dispersed in water. As can be seen from Fig. 7, exposure to UV light can highlight the effectiveness of the process to give desired properties. The emission spectrum of flower-like NPs in water shows the presence of a strong peak at about 515.88 nm, which is indicative of the FITC presence in the silica network^39^.

(b) Dispersion of nanoparticles in finishing inks

**Figure 8:**
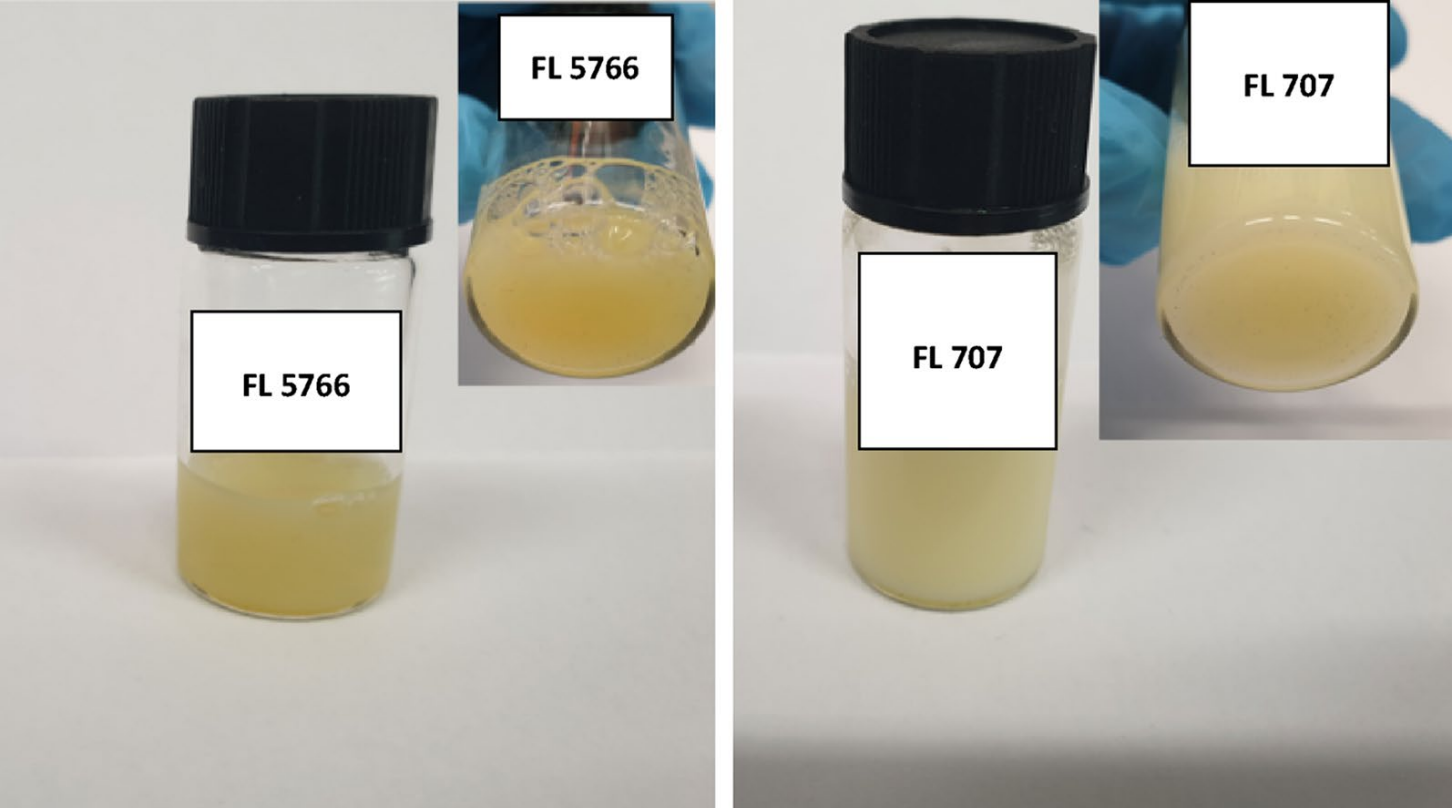
Nanoparticles flower-like in chemicals 5766 and 707.

The synthetic flower-like nanoparticles were also dispersed in water-based chemicals commonly used for leather finishing, Fig. 8. In particular, in Fig. 8 two photos showing well-dispersed nanoparticles in the 5766 and 707 finishing inks are reported.

#### Chemical-physical characterization of the leathers

##### SEM analysis

**Figure 9:**
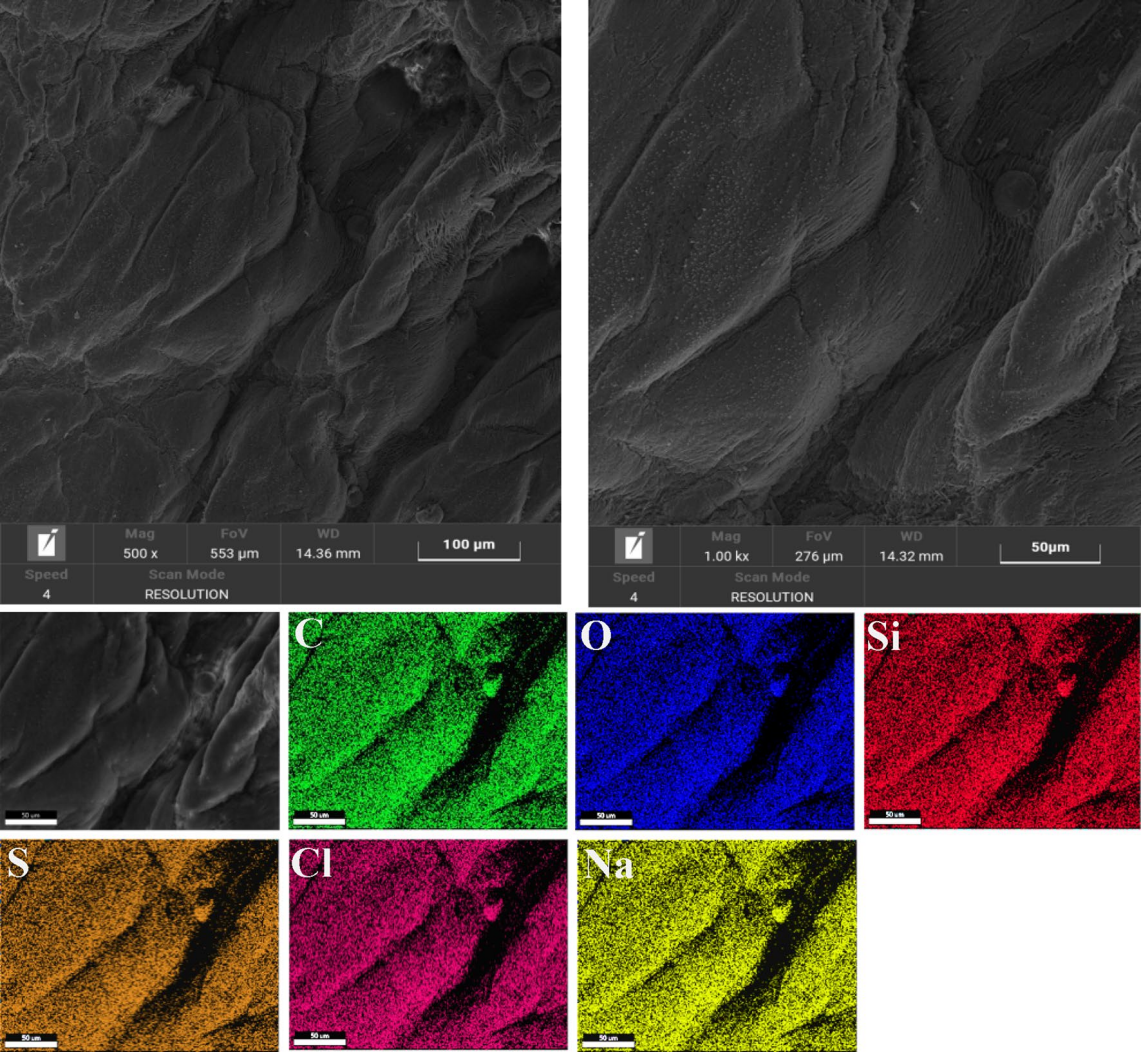
SEM images analysis of the Sample W.

**Figure 10:**
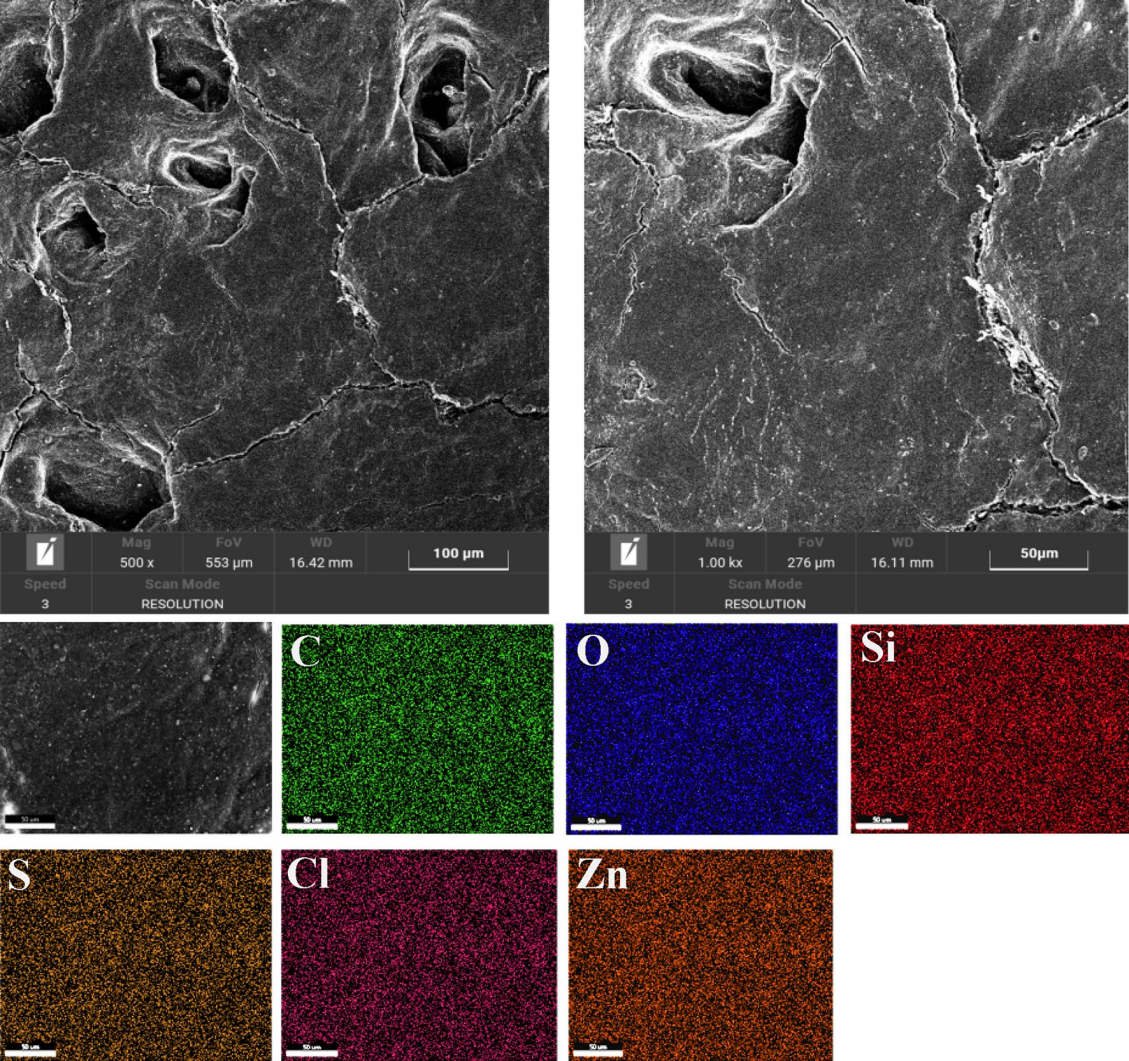
SEM images analysis of the Sample P.

The images obtained from SEM analysis in Fig. 9 (Sample W) and Fig. 10 (Sample P) showed that the surface of untreated leather is typically smooth. Moreover, the EDS maps of sample W and sample P show carbon and oxygen elements, which are attributed essentially to their organic composition. The presence of low amounts (< 0.5%) of sodium, silicon, sulfur, chloride, and zinc can be ascribed to the chemical products used in leather processing.

**Figure 11:**
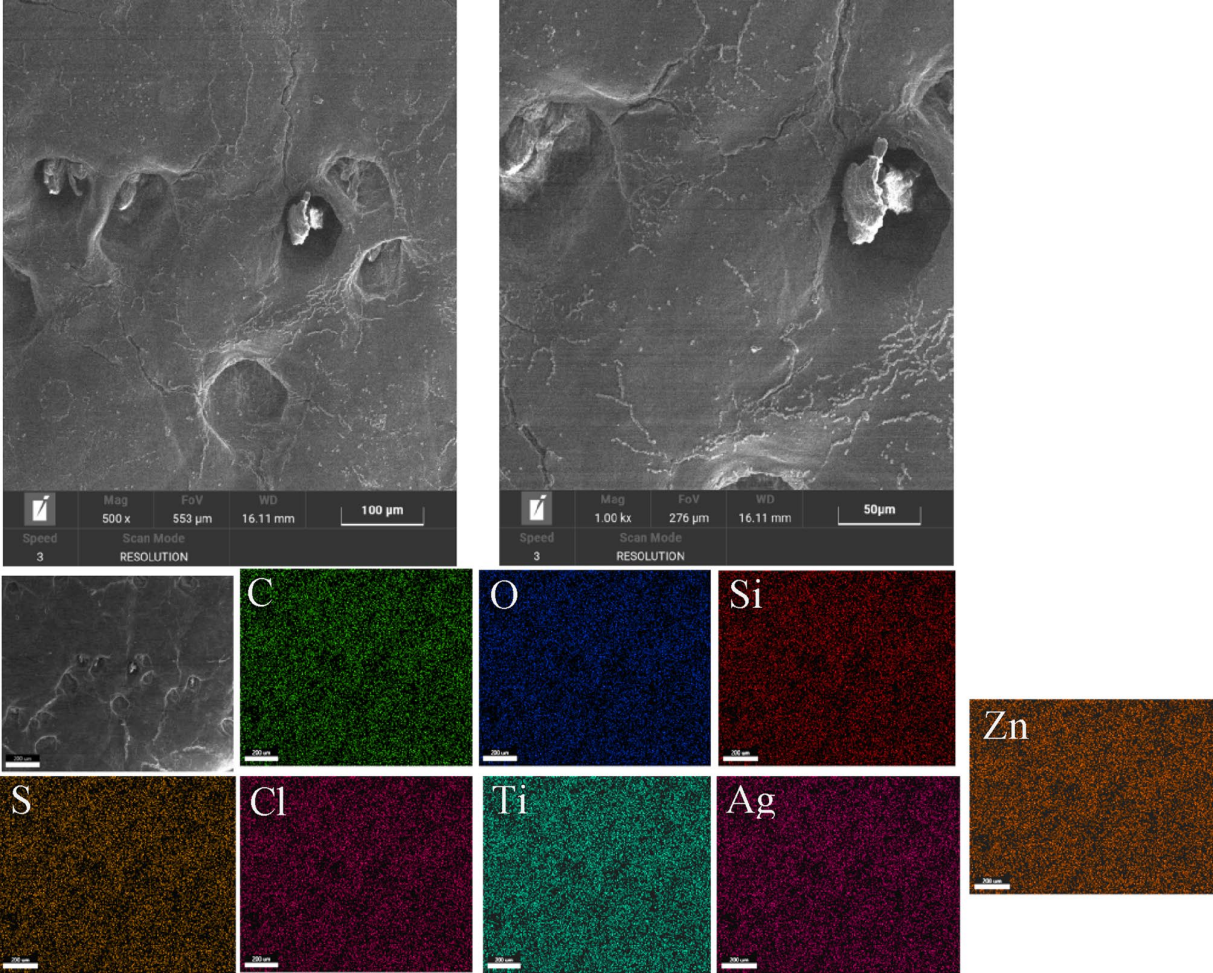
SEM images analysis of the sample A.

**Figure 12:**
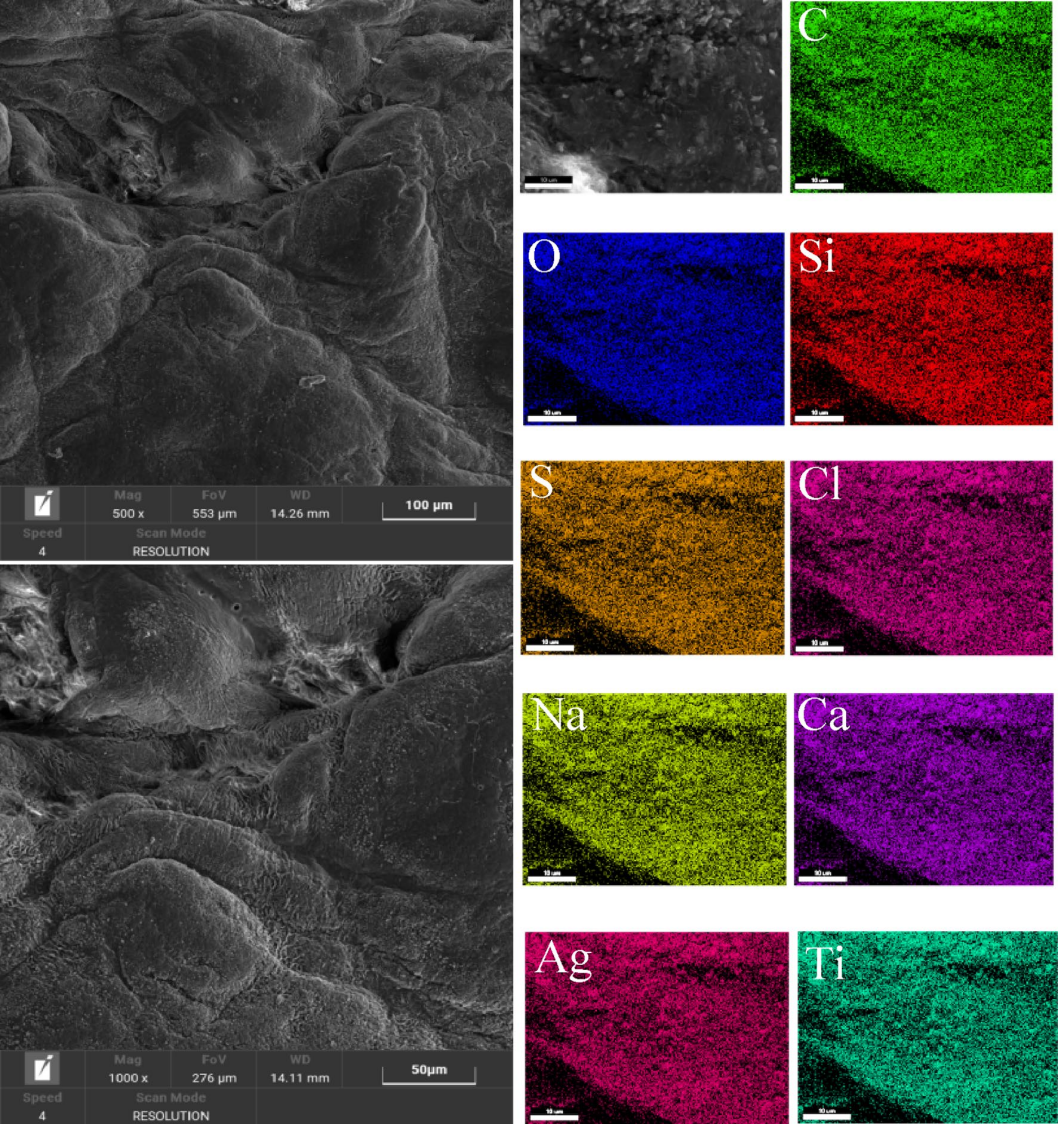
SEM images analysis of the sample C.

The SEM analysis after finishing with flower-like NPs (Figs. 11 and 12) shows the morphology of the leather surfaces, uniformly covered by the different elements. In particular, chemical analysis confirmed the presence of Ag, Ti, and Si together with C, and O, indicating the good and uniform distribution of the nanoparticles. NPs on the surface.

##### XPS analysis

The elemental compositions and chemical status of leather with and without finishing have been analyzed by XPS. In particular, Fig. S1a shows the XPS analysis of samples W and D. The XPS spectra survey of sample D shown in Fig. S1a exhibits clear signals for the elements C, N, O, Si, Ti, and Ag. Figure S1b displays the deconvoluted C 1 s peaks of the sample W, revealing the presence of the four peaks binding energies at 285.0 eV corresponding to C–C/C–N, at 287 eV corresponding to C–O/C–N, at 288 eV corresponding to C=O and 290 eV corresponding to COO^−^^40,41^. In contrast, the C 1 s spectrum of sample D shows a substantial difference, which suggests the potential interaction of carboxyl and carbonyl groups with the FL_10_ NPs on the surface of the leather^42,43^. Moreover, a slight shift in the high binding energy of N 1 s in sample D is due to the hydrogen bonding of FL nanoparticles with the leather surface^43^. Furthermore, the high-resolution XPS spectra of Ti 2p, Ti 2p_3/2_ (458.7 eV), and Ti 2p_1/2_ (464.1 eV), peaks are shown in Fig. S2a, which are assigned to the Ti^4+^ oxidation state^44^. It is low visible in the presence of Ag in the XPS survey of sample D, due to the concentration of Ag which is extremely low (see Fig. S1a). Figure S1b shows the high XPS spectra of Ag 3d. In particular, Ag 3d_3/2_ and Ag 3d_5/2_ peaks at the binding energy of 368.1 and 374.3 eV which are characteristics of metallic Ag 3d states^45^. The presence of Si in sample D can be better observed at 103.3 eV^46^, the spectrum is illustrated in Fig. S2c.

##### Thermogravimetric analysis (TG-DTG)

**Figure 13:**
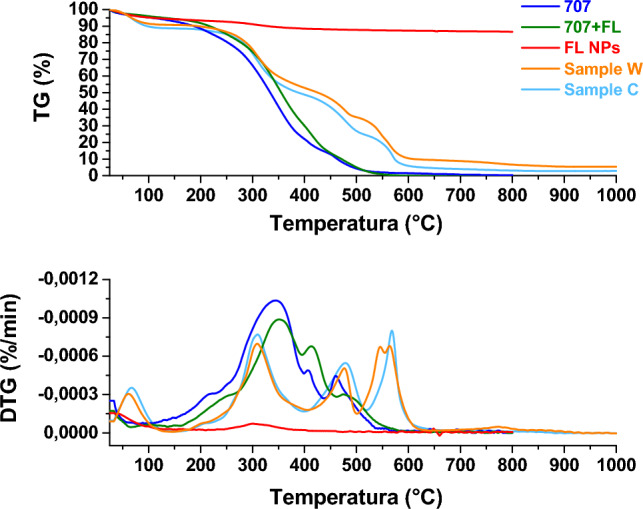
Thermogravimetric analysis was performed on flower-like NPs (red profile), chemicals 707 (blue profile), chemicals 707 with flower-like NPs (green profile), sample W (orange profile), sample C (cyan profile).

**Figure 14:**
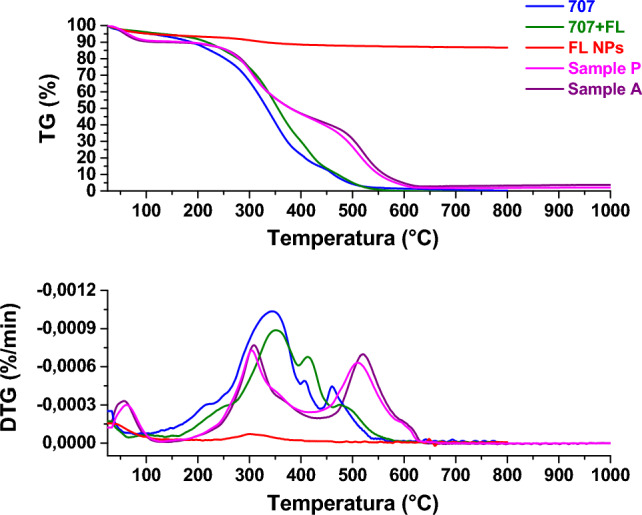
Thermogravimetric analysis was performed on flower-like NPs (red profile), chemicals 707 (blue profile), chemicals 707 with flower-like NPs (green profile), sample P (magenta profile), sample B (violet profile).

**Figure 15:**
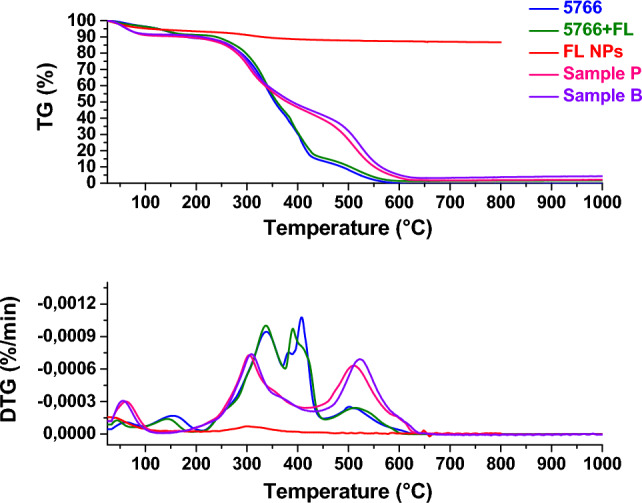
Thermogravimetric analysis was performed on flower-like NPs (red profile), chemicals 5766 (blue profile), chemicals 5766 with flower-like NPs (green profile), sample P (magenta profile), sample B (violet profile).

**Figure 16:**
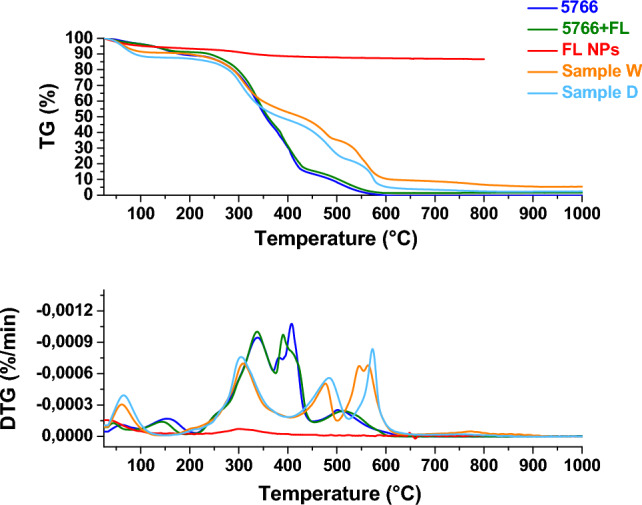
Thermogravimetric analysis was performed on flower-like NPs (red profile), chemicals 5766 (blue profile), chemicals 5766 with flower-like NPs(green profile), sample W (orange profile), sample D (cyan profile).

The samples were analyzed by thermogravimetric analysis to assess weight loss as temperature increases. Figures 13, 14, 15 and 16 show the thermogravimetric profiles of the different samples, reported in Table 1, for comparison. The profiles of the 707 and 5766 inks, flower-like NPs, and NP-added inks are also reported. In particular, the presence of nanoparticles inside the inks determines a slight increase in the stability of the polymers, see and compare the blue and green profiles in the different figures. The presence of the flower-like nanoparticles in the finishing polymers, also, determines increased stability for the leathers covered with the thin finishing layer, see the profiles of the W and C, P and A, P and B, W and B in Figs. 13, 14, 15 and 16, respectively. Concerning this last aspect, the upshift of the DTG peaks of the leather-coated samples regards the weight losses at higher temperatures, which are mainly related to the oxidation of organic hydrocarbon chains and, to the degradation, the final, of the soft segment of the polymer^4^. Although flower-like nanoparticles are present in small amounts, and only in the finishing layer, their presence, probably mainly due to the SiO_2_ component^3,4,17^, causes an increase in the stability of the leathers. After all, the finishing layer has very little influence in gravimetric terms on the weight of different leather samples^47^.

#### Characterization of leather properties

- Self-cleaning properties under UV–vis light

Methylene Blue (MB) discoloration under UV exposure was detected over time for the leathers finished with the flower-like NPs and the control sample by photography (Table 2). It is possible to observe the discoloration that the finished leathers show under UV exposure in comparison with the control sample. Under UV light, degradation of the MB stain occurs, becoming evident in 15 h. The leather samples finished with flower-like NPs are completely clean, after 15 h of exposure. This result can be attributed to the photocatalytic reactivity of the binary TiO_2_ in combination with silica. A role for silver^48^, in averting the recombination of electron-hole pairs thanks to the transfer of photo-excited electrons to the higher conductive Ag metallic juncture NPs, cannot be neglected, too. In particular, the comparison with the behavior of titania-added NPs alone, which are unable to degrade MB in 15 h, highlights the character of the other components, indicating a role for silica heterojunction of our NPs in increasing adsorption of pollutants^49^ and more efficient separation of the holes, being transported to the TiO_2_ surface, and thus lowering the recombination rate of the electron-hole pairs for the TiO_2_^50^. Moreover, the SiO_2_ addition increases the amount of water and hydroxyl groups adsorbed on the surface, improving the hydrophilic and photocatalytic properties^51–53^.

**Table 2:** The self-cleaning properties of the leather surface under UV light exposure.

Time	Control	Sample D
Initial stained		
After 3 h of UV light exposure		
After 15 h of UV light exposure		

b) Aging tests

**Figure 17:**
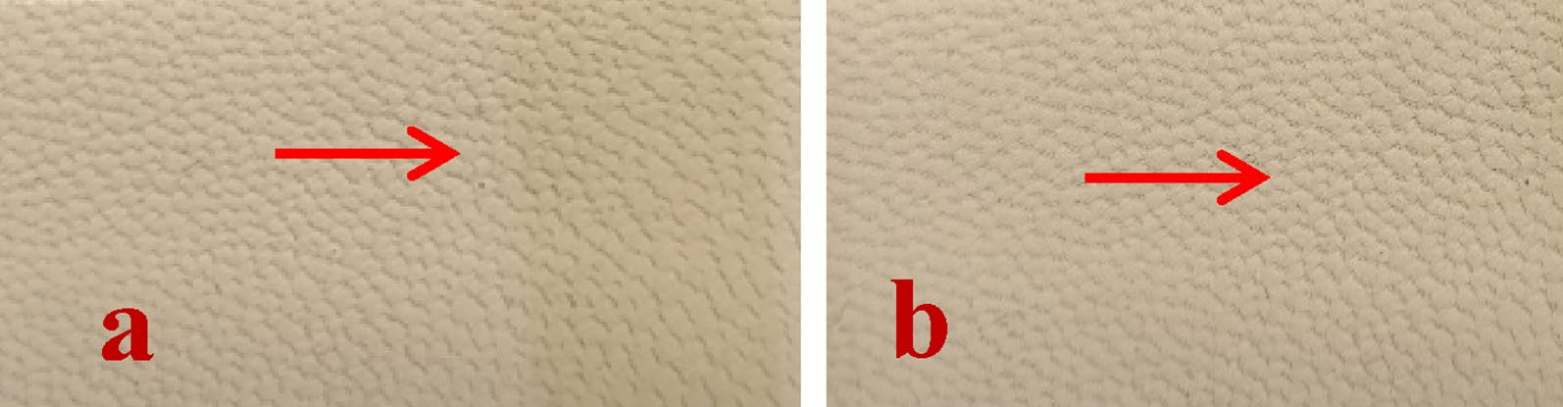
Leather samples: (**a**) finished with a polyurethane formulation (5766); (**b**) sample D, after an aging test (ISO 105-B02), see the area indicated by the red arrows, in comparison with untreated areas.

The aging tests in artificial light, conducted on the leather samples reported in Fig. 17, show that the sample finished with 5766 ink alone, presents a marked yellowing, see the area indicated by the red arrows in Fig. 17a. On the other hand, the tests of exposure to artificial light on samples finished in the presence of flower-like nanoparticles dispersed in 5766 ink allow us to observe an evident improvement in the behavior after 72 h of exposure.

Furthermore, the accelerated aging tests were also performed on other leather samples.

Specifically, Table 3 shows photos of leather samples after 24 h and 48 h of exposure to a xenon lamp at a temperature of 50 °C. For comparison, a portion of the sprayed samples were covered during the test to avoid exposure. From the aging tests, after 24 h and 48 h, the influence of the flower-shaped nanoparticles on the color change can be observed. In particular, it is possible to note how the finishing with NPs flower-like improves the resistance to fading of the leather. This result was confirmed by the color fastness test (Table 4), performed according to ISO 17228. The grayscale values, which are indicative of the color discharge, 1 = significant variation (worst rating) and 5 = no variation (best rating), evidence the very good color fastness recorded for the NPs functionalized samples.

**Table 3:** Aging test according to ISO 105 B02 standard.

Leather sample	Exposure time
Leather with 5766 finishing (white)	
Sample D	
Leather with 707 finishing (white)	
Sample C	
Leather with 5766 finishing (pink)	
Sample B	
Leather with 707 finishing (pink)	
Sample A

**Table 4:** Accelerated aging according to ISO 17228.

Sample	Gray scale chromatic difference between the various grey pairs at time 0 and after 72 h^a^
Leather with finishing 707	4
Leather with finishing 707 + FL	5
Leather with finishing 5766	4
Leather with finishing 5766 + FL	5

^a^1 = significant variation (worst rating) and 5 = no variation (best rating).

The shrinkage of the specimens was evaluated by measuring their dimensions before and after the execution of the aforementioned tests, too. It was found that the presence of the flower-like nanoparticles in the finishing layer significantly limited the effects of curvature/surface morphological alteration due to heat treatment (Table 5).

**Table 5:** Footwear/leather goods samples according to ISO 17228.

Sample	Initial test	After 72 h
Leather with finishing 5766		
Sample B		

The color change and durability of the leather is one of the parameters of primary importance as it expresses the fading of the color and therefore possibly transfer to the adjacent fabrics, this phenomenon can also occur in the leather interiors of cars which are subject to aging due to the bright electromagnetic radiation from the sun and the heat that this radiation induces. Accelerated aging tests were conducted on leather samples intended for the automotive sector, too. Table 6 reports the test results, which show that the ΔE value (variation in terms of color), together with all other color parameters, is lower for the samples finished with flower-like NPs than for the untreated sample. The purpose of the SAE J2412 standard is to define a method to simulate extreme environmental conditions that can occur inside the vehicle due to sunlight, heat, and humidity, to predict the behavior of the interior materials car. The samples are tested inside xenon arc chambers for a pre-set time, which can vary, i.e. it is possible to run the test for a pre-set number of hours or a pre-set number of Kj. At the end of the test, the samples will be extracted from the test chamber, photographed highlighting any defects or anomalies that may have appeared, and examined to express a judgment of conformity. Color difference values (∆E, ∆H, ∆C, ∆L, ∆a, ∆b) in CIELAB units are obtained by instrumentally measuring the reference fabrics before and after a specified amount of radiant exposure.

**Table 6:** Aging tests according to SAEJ 2412: evaluation of color fastness on leather samples intended for the automotive sector.

Characteristics	Test method	Requirements	U.M.	Control sample	Sample B
				Results	Results
To artificial light 225 $\frac{{\text{KJ}}}{{{\text{m}}}^{2}}$	SAEJ 2412 (acc. MS-JK 4000)	∆E ≤ 3.0 (for closed saloon car)	∆E	1.02	0.76
		Report ∆H, ∆C and L,a,b data	∆H	∆H:0.24	∆H:0.04
			∆C	∆C:0.13	∆C:0.08
			∆L	∆L:0.98	∆L:0.76
			∆a	∆a:0.04	∆a:0.01
			∆b	∆b:0.27	∆b:0.09
To artificial light 601 $\frac{{\text{KJ}}}{{{\text{m}}}^{2}}$	SAEJ 2412 (acc. MS-JK 4000)	TBR	∆E	1.03	0.60
		Report ∆H, ∆C and L,a,b data	∆H	∆H:0.48	∆H:0.04
			∆C	∆C:0.42	∆C:− 0.14
			∆L	∆L:0.81	∆L:0.58
			∆a	∆a:0.09	∆a:0.00
			∆b	∆b:0.63	∆b:0.14

Color is one of the most important visual characteristics of the finishing film, in terms of its direct impact on consumer acceptance. Typically, lightness (L*), and yellowness (b*) parameters can increase with increasing TiO_2_ NP content, whereas the redness (a*) parameter decreases^54^. LSPR (localized surface plasmon resonance) property of silver nanoparticles, can impart a yellow/brownish color to the leather surface [17)]. The reduction of the photofading can be obtained by incorporation of appropriate additives into the dyed or pigmented medium which will remove the harmful light energy (absorption) by some effective means. Many pigments can act as photo stabilizers, either reflecting and/or absorbing the damaging incident light, showing antioxidant and UV-stabilized behavior. The results reported in Tables 3, 4, 5 and 6 can be attributed to the presence of the TiO_2_ rutile and SiO_2_ in our flower-like NPs. The rutile form of titanium dioxide is of commercial interest for polymer color stabilization. On the other hand, although, anatase is markedly photosensitive in degrading the polymer, it is stabilized by surface covering^55^. Moreover, silica can considerably inhibit pigment photoactivity and confer good stabilization^56^.

c) Antibacterial tests

The analysis of the results shown in Table 7 evidence improved antibacterial properties in the presence of flower-like NPs in the finishing layer. This result is certainly attributable to the Ag antibacterial activity^20,57–59^ and Ag/TiO_2_ heterojunction^20,60,61^ together with the antimicrobial activity exhibited by TiO_2_ in synergy with SiO_2_. Since the catalytic activity of TiO_2_–SiO_2_ mixed oxides results enhanced by increased surface area, and formation of additional distinctive active sites, such as the Ti-O-Si and Si-O-O bridging bonds at the interface and the TiO_2_–SiO_2_ bond^62^.

**Table 7:** Results of antibacterial tests on pink leathers (TTC/MALT dipslide tests).

Sample	CFU/mL MALT			CFU/mL TTC		
	24 h	48 h	72 h	24 h	48 h	72 h
Leather with 707 finishing	//	10^4^	10^4^	< 10^2^	< 10^3^	10^3^
Leather with 707 + FL_10_ finishing (sample A)	//	< 10^2^	< 10^3^	< 10^1^	< 10^2^	< 10^2^
Leather with 5766 finishing	< 10^3^	10^4^	10^4^	< 10^2^	10^3^	10^4^
Leather with 5766 + FL_10_ finishing (sample B)	//	< 10^3^	< 10^3^	< 10^1^	10^2^	10^3^

d) Abrasion resistance tests and wear and micro-scratch measurements

**Figure 18:**
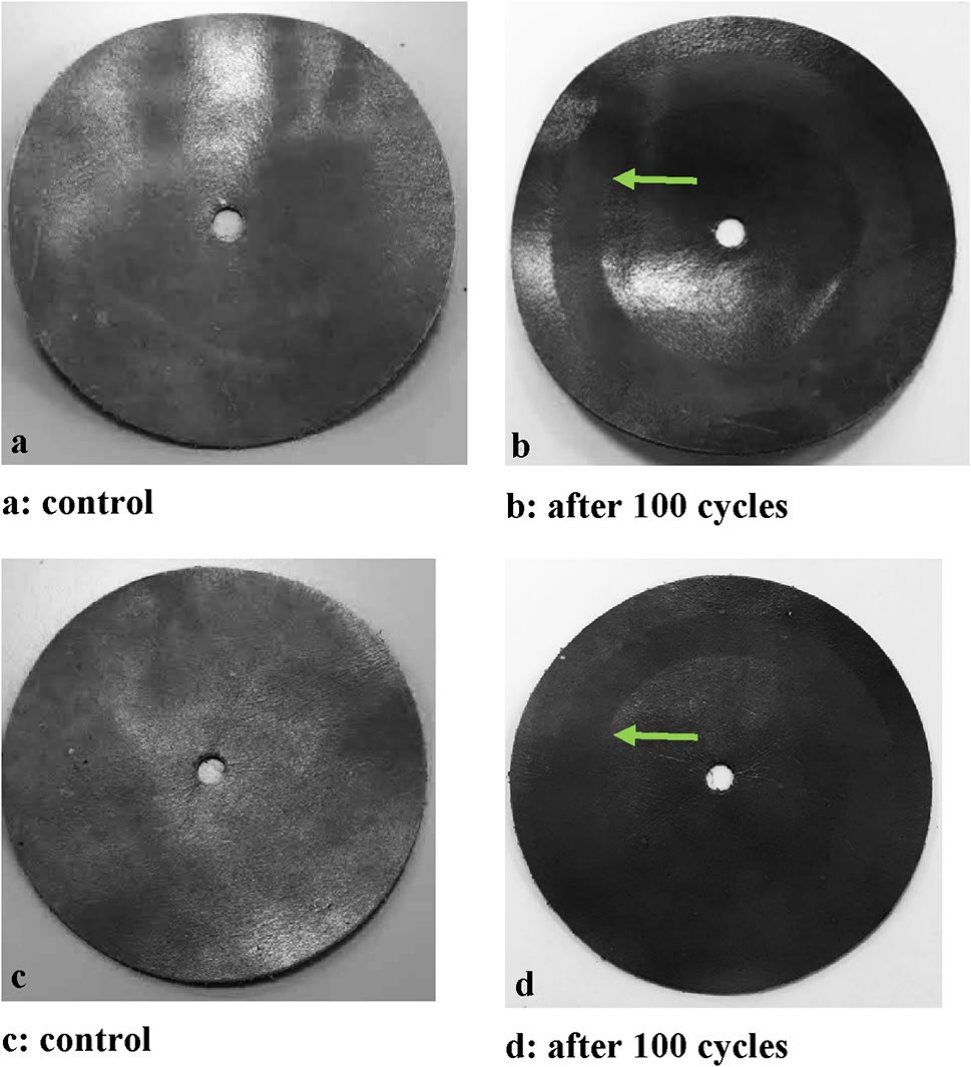
The leather samples tested for abrasion resistance (Table 8): (**a**) leather sample with 707 finishing (before testing); (**b**) leather sample with 707 finishing (after 100 cycles); (**c**) Sample A (before testing); (**d**) Sample A (after 100 cycles).

Abrasion resistance tests were carried out to test the mechanical resistance of the nanoparticles added finishing layer. The results show that the presence of the NPs in the surface finishing layer is capable of improving the performance of the treated samples, compare the gray scale differences and see the areas indicated by the green arrows in Table 8 and Fig. 18, respectively.

**Table 8:** Abrasion resistance tests (ISO 17076).

Sample	Gray scale chromatic difference between the various grey pairs at time 0 and after 100 cycles^a^
Leather with finishing 707	3
Leather with finishing 707 + FL_10_ (sample A)	4

^a^1 = significant variation (worst rating) and 5 = no variation (best rating).

Moreover, Table 9 shows the resistance to rubbing and abrasion tests of leathers finished with flower-like NPs in comparison with a standard finished sample. The results show a more than significant improvement, with an increase in resistance more than doubled. This result is ascribed to the presence of flower-like NPs, which determine an increase in the density of material in the roughness of the covered leather surface, i.e. the nanoparticles well dispersed in the finishing polymer effectively fill the micropores or pinholes in the coating, thereby reducing the defect density of the coating itself, improving fastness to dry and wet rubbing^63,64^.

**Table 9:** Abrasive tests on leather finishing: control sample and with flower-like NPs.

			Control sample	Leather with flower-like finishing
Characteristics	Test Method	Requirements	Results	Results
To rubbing with alcohol	FCA 50444	TILL END OF LIFE ≥ 3 bleeding on cloth	Beginning break 10 CYCLES	Beginning break 50 CYCLES
Martindale ball plate	DIN EN ISO 17076-2	TILL END OF LIFE	Beginning break 1500 CYCLES	Beginning break 3000 CYCLES
TABER TEST_CS-10, 10 N	ISO 17076-1	TILL END OF LIFE Slight traces of abrasion are permitted Not permissible: cracks in the finish such that the crust leather surface is visible	Beginning break 1000 CYCLES	Beginning break 2000 CYCLES

Standards in the Table column 2; U. M. Cycles.

**Figure 19:**
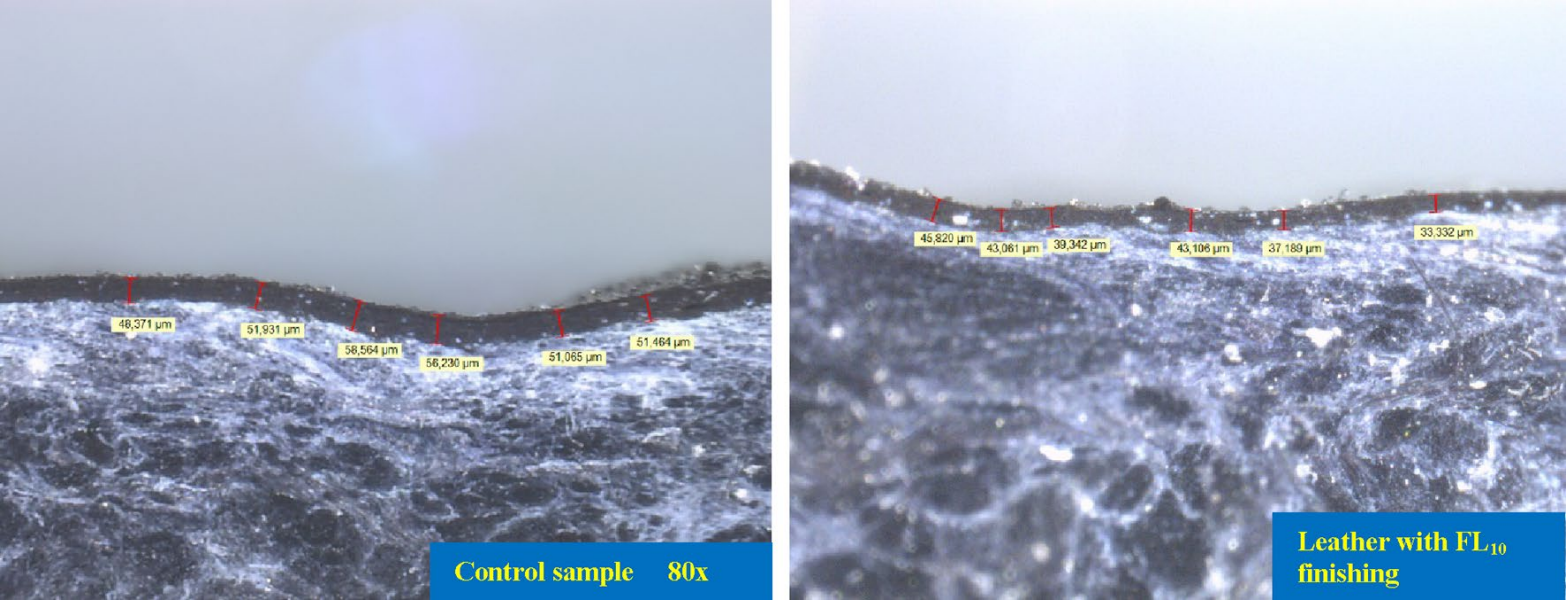
Cross section images of leathers finished without and with flower-like nanoparticles, over testing at 80×.

What is more, additional surface tests were performed to explore the possibility of reducing the thickness of the finishing polyurethane film, which means a reduction in covering material and therefore costs, as well as improved features of the surface and leather which appears more natural to eyes and touch. Abrasive resistances, comparable to those reported in Table 9 column 4, were observed for a leather sample, finished in the presence of flower-like NPs, at a 20% reduced finishing thickness, see Fig. 19.

**Figure 20:**
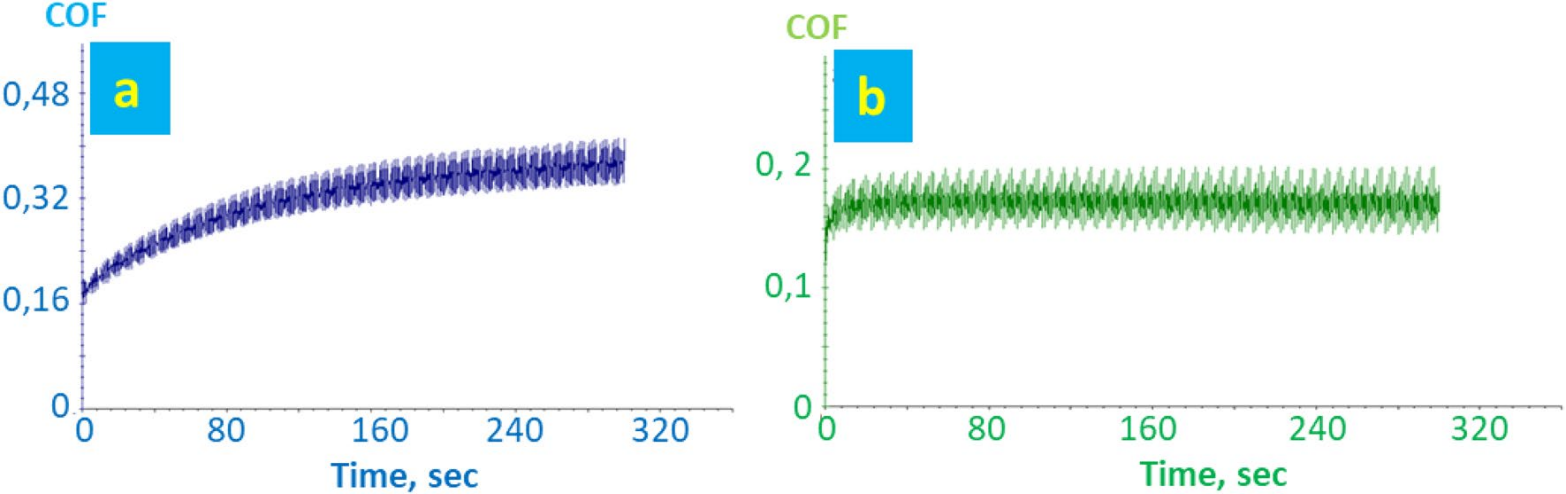
Wear measurement of leather with finishing 707: (**a**) finished face. Wear measurement of leather sample A: (**b**) finished face.

**Figure 21:**
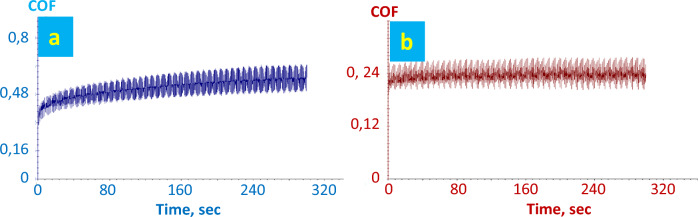
Wear measurement of leather with finishing 5766: (**a**) finished face. Wear measurement of sample B: (**b**) finished face.

Leather samples before and after finishing were characterized for wear and micro-scratching resistance. Figure 20 shows the trend of coefficient of friction versus time for the leather samples coated with 707 chemical finishing and NPs flower-like in 707 finishing. Figure 21 shows the trend of COF regarding the leather samples coated with a 5766 finishing and NPs flower-like in a 5766 finishing. Lower values of COF to wear indicate better material response to surface friction. In particular, in the case of the FL-added finishing layer, the COF stays constant under increasing time, indicating a remarkable resistance of the finishing layer. In this regard, it has been reported that SiO_2_ NPs are one of the most suitable nano-reinforcement nanomaterials to improve the wear resistance of coatings^65^.

**Figure 22:**
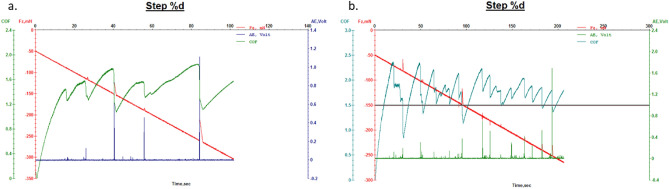
Microscratch measurement of leather with finishing 707: (**a**) 707 alone; (**b**) sample A.

**Figure 23:**
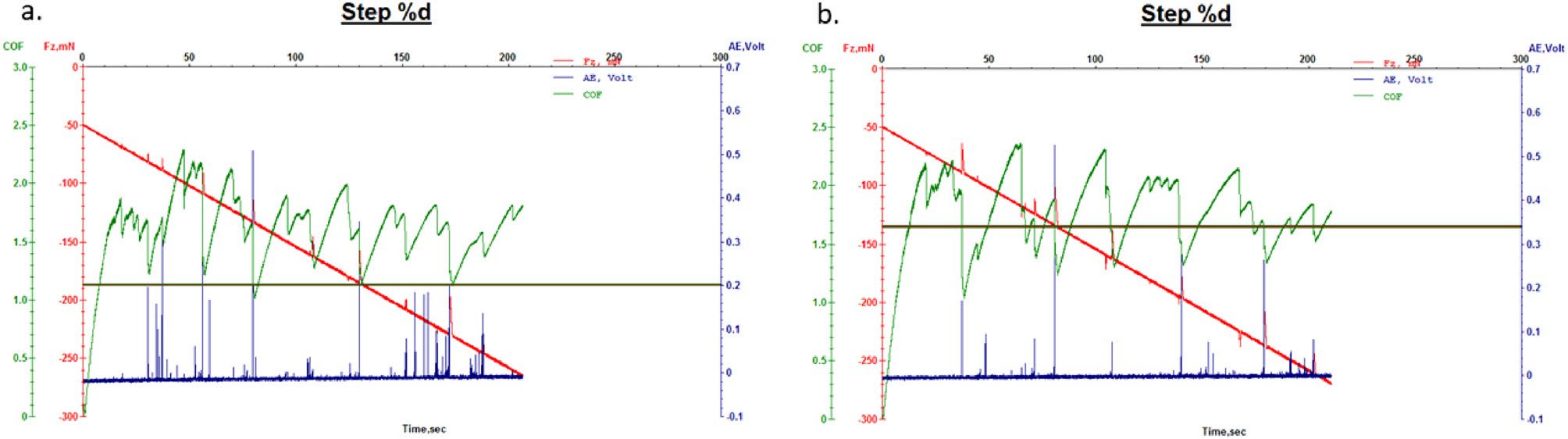
Microscratch measurement of leather with finishing 5766: (**a**) 5766 alone; (**b**) sample B.

Next, Figs. 22 and 23 show the results of micro-scratch tests for leather samples finished with finishing chemicals 707 and, flower-like NPs added 707; and for leather samples finished with finishing chemicals 5766 and, flower-like NPs added 5766, respectively. In this case, improved tangential forces (Fx) in the presence of FL-added finishing layers determined increased COF which means better resistance to micro-scratching for the NPs based-finishing.

The excellent performance observed in the case of our FL NPs functionalized finishing layers, also if compared with other reported results^4,17,60^, are certainly partially ascribable to the morphology and size of the flower-like NPs. Indeed, the strong coupling between the particle size, local surface roughness, surface energy, and elastic constant of contacting materials are the reasons for very large variations in adhesion^66^.

e) Evaluation of the contact angle and absorption dynamics

As part of this work, the ability of functionalized finishes to confer higher leather protection against water was evaluated. Water repellence was very useful for leather and derived products, for self-cleaning properties and protection of leather mechanical strength, and thus product stability and shape, which can be easily damaged by water penetration^58^.

**Figure 24:**
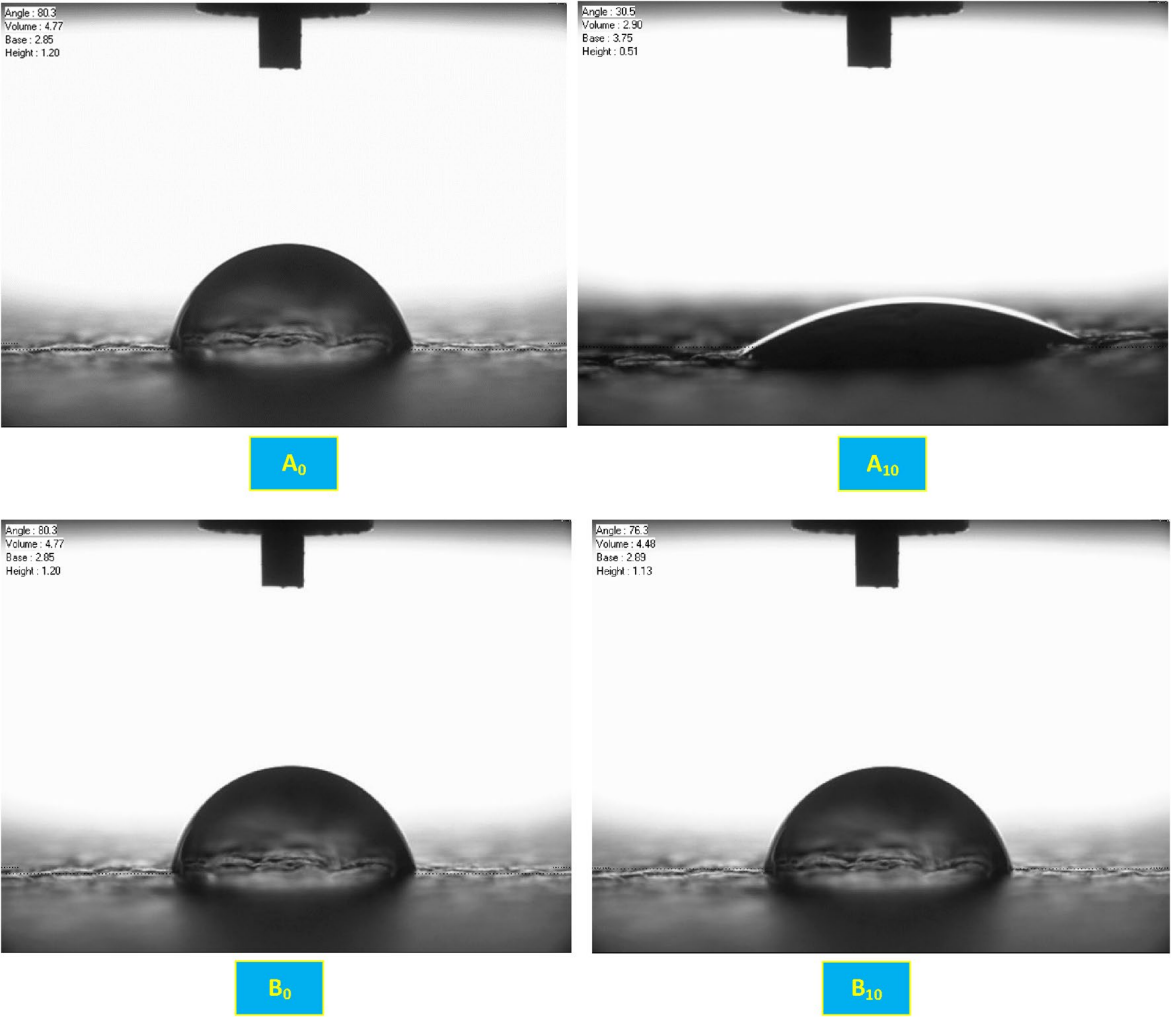
Determination of the contact angle at the moment of impact, time 0, and after 10 s, for a reference sample, Figure A0 and A10; and sample with flower-like finishing, Figure B0 and B10.

In particular, the water contact behaviour was investigated by contact angle analysis, Fig. 24. The contact angle analysis is a simple experiment where the image of a water droplet on a given substrate is captured and the angle that traces the air–water to water–substrate interface can be observed from the origin of the air–water-substrate contact point at the edge. For this purpose, a sample finished with only polyurethane-based 5766 ink, and a sample treated superficially with FL nanoparticles in polyurethane (sample B), are shown in Fig. 24. It can be observed a significant increase in the performance, compared to Fig. 24A10 and B10, showing that the water drop contact angle is practically unchanged after 10 sec in the case of nanoparticles containing finishing.

f) Fluorescence leather

**Figure 25:**
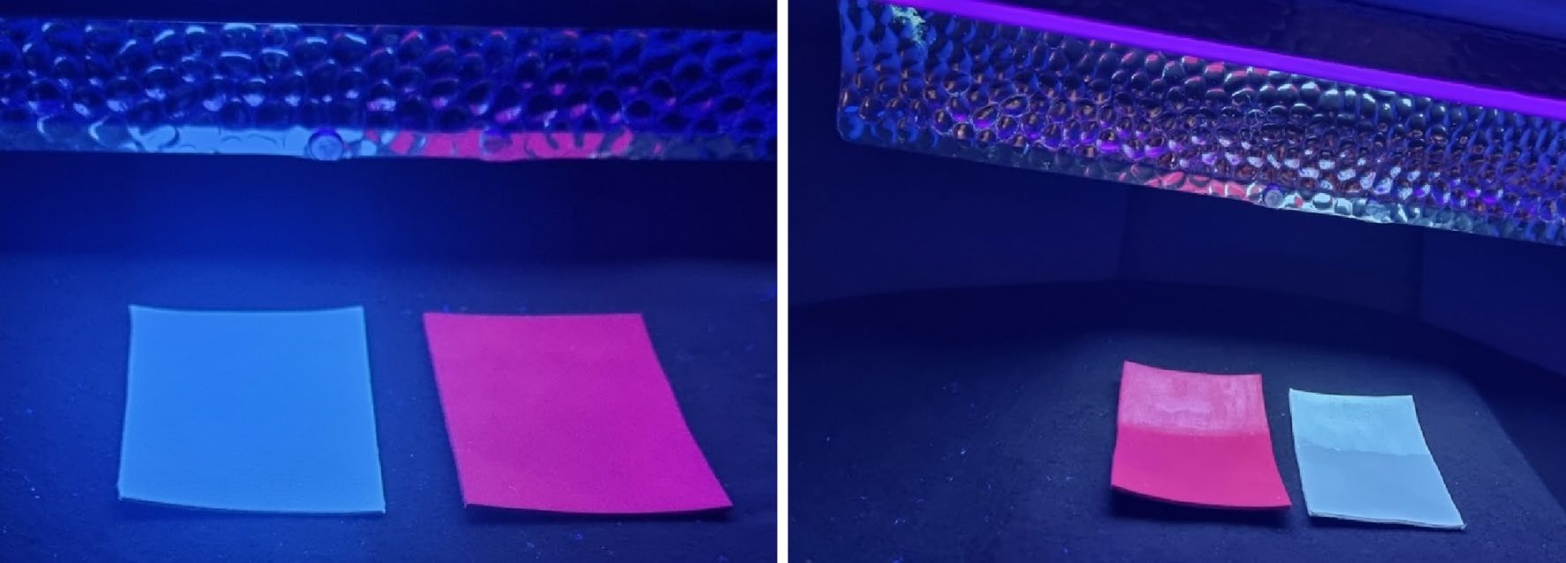
Photographs: of samples B and D before finishing; and, of samples B and D after finishing under UV lamp (λ = 365 nm) illumination.

Finally, the fluorescence behaviour, which can be useful in protecting products from counterfeiting, was verified on finished leather samples using a UV lamp. Figure 25 shows some functionalized leather samples (white and pink) under UV illumination, evidencing that half of the samples are fluorescent.

## Conclusions

The synthesized flower-like nanoparticles were characterized both from a chemical-physical and morphological point of view. Specifically, TEM analysis revealed the formation of nanoparticles with structural similarities with flowers of uniform size (about 65–70 nm in diameter), made up of a TiO_2_ core (“pistil”) and various “petals”, of smaller dimensions, silica, and silver NPs. The flower-like NPs show high dispersibility and stability in hydrophilic environments.

Although flower-like nanoparticles are present in small amounts, and only in the finishing layer, their presence, probably mainly due to the SiO_2_ component, determines increased thermal stability.

Very interesting self-cleaning properties, as assessed by MB stain degradation under UV light, were observed. They are found to be completely clean after 15 h of exposure. This result can be primarily attributed to the photocatalytic reactivity of the binary TiO_2_/silica; i.e., silica heterojunction for increasing adsorption of pollutants; and, more efficient separation of the holes, being transported to the TiO_2_ surface, lowering the recombination rate of the electron–hole pairs for the TiO_2_. A role of silver, in averting the recombination of electron–hole pairs thanks to the transfer of photo-excited electrons to the higher conductive Ag metallic juncture NPs, cannot be neglected, too. Color and color fastness enjoy LSPR of the NPs and rutile stabilization in the presence of anatase-covered surfaces. Antibacterial tests highlight improved antibacterial properties in the presence of flower-shaped NPs in the finishing layer. This result is certainly attributable to the antibacterial activity of Ag and the Ag/TiO_2_ heterojunction together with the antimicrobial activity exhibited by TiO_2_ in synergy with SiO_2_. Furthermore, leather samples coated with flower-like nanoparticles were subjected to abrasion, wear, and scratch resistance tests, since abrasion is among the most unwanted but inevitable defects of using any leather product subjected to rubbing and scraping every day. The leathers finished with flower-like NPs show a significant improvement in abrasion resistance, exhibiting a more than doubled increase in resistance. This is due to the presence of flower-shaped NPs well dispersed in the finishing polymer, which effectively fills the micropores of the coating, thus reducing the density of defects in the coating itself and improving fastness to dry and wet rubbing. A reduction in finishing thickness of 20%, maintaining the best performances required for luxury automotive sector leathers, was allowed. This means a reduction in covering material and therefore costs, as well as improved features of the surface and leather, which appears more natural to the eyes and touch. Finally, fluorescence behavior, which can be useful for protecting products from counterfeiting, is tested and confirmed.

In this study, the superior efficacy of the finishing coating towards a series of multifunctional properties was obtained by adding mass-produced nanoparticles, enjoying different properties and enhancement/amplification of these properties due to the heterojunctions between different species.

Furthermore, the fluorescent behaviour of the prepared finishing coating can be used as an anti-fraud method for recognizing leather from eco-leather. The prepared fluorescent ink is, also, environmentally friendly and can be widely used in anti-fraud fields for footwear and automotive industries. In summary, nanomaterials, applied in the finishing layer, can confer different properties, such as increased thermal stability, protection against micro-organisms, as well as self-cleaning properties, etc.…, leading to increased leather versatility. The close cooperation between industry and scientific research, allowing the performance evaluation in real conditions, and following deposition and standards used by industries, with particular attention to the feasibility of the operations also from management and sustainability point of view, makes these results much more significant and perspective. Safety and cost assessments, also studied and evaluated, will be reported in a further paper.

### Supplementary Information

Supplementary Figures.

## Data Availability

The datasets used and/or analysed during the current study available from the corresponding author on reasonable request.
