# Supplementary material for: Multifunctional leather finishing vs. applications, through the addition of well-dispersed flower-like nanoparticles
Source: Sci Rep. 2024 Jan 25;14:2163. doi: 10.1038/s41598-024-51775-4 (PMC10811342; doi:10.1038/s41598-024-51775-4)
Supplement: Supplementary file 1 — Supplementary Figures. [file 41598_2024_51775_MOESM1_ESM.docx]

**Supporting Material**

**Multifunctional leather finishing vs. applications, through the addition of well-dispersed flower-like nanoparticles**

Francesca Fierro^a,c°^, Mariagrazia Iuliano^b,c°^, Claudia Cirillo^a,c°*^, Claudia Florio^d^, Gaetano Maffei^e^, Andrea Loi^f^, Todor Batakliev^g^, Renata Adami^a^, Maria Sarno^a,c^

^a^Department of Physics “E.R. Caianiello”, University of Salerno, Via Giovanni Paolo II, 132 - 84084 Fisciano (SA), Italy

^b^Department of Industrial Engineering, University of Salerno, Via Giovanni Paolo II, 132 - 84084 Fisciano (SA), Italy

^c^Centre NANO_MATES, University of Salerno Via Giovanni Paolo II, 132 - 84084 Fisciano (SA), Italy

^d^Stazione Sperimentale per l'Industria delle Pelli e delle materie concianti - SSIP (Italian National Leather Research Institute), Comprensorio Olivetti, Via Campi Flegrei, 34 - 80078 Pozzuoli (NA), Italy

^e^ Conceria DMD SOLOFRA Spa, Via Celentane, 9 - 83029 Solofra (AV) - Italy

^f^ Mario Levi Italia s.r.l., Via Arzignano, 130 - 36072 CHIAMPO (VI) - Italy

^g^Open Laboratory on Experimental Micro and Nano Mechanics (OLEM), Institute of Mechanics, Bulgarian Academy of Sciences, Acad. G. Bonchev Str., Block 4, 1113 Sofia - Bulgaria

°These authors contribute equally

Figure S1 XPS wide scan spectra of sample W and D; High-resolution XPS spectra of C 1s for sample W (b) and sample D (c).

Figure S2 High-resolution XPS spectra of Ti (a); Ag (b); and Si (c) for Sample D.
